# The therapeutic efficacy of radical resection for hepatocellular carcinoma varies markedly by tumor location: a retrospective real-world study

**DOI:** 10.3389/fphar.2025.1674998

**Published:** 2025-12-03

**Authors:** Xu Feng, Yi-Qiu Wei, Jia-Rui Liu, Zheng-Rong Shi, Kai Chen, Yong-Shuang Lv

**Affiliations:** 1 Department of Hepatobiliary Surgery, The Affiliated Yongchuan Hospital of Chongqing Medical University, Chongqing, China; 2 Department of Hepatobiliary Surgery, The First Affiliated Hospital of Chongqing Medical University, Chongqing, China; 3 Department of Imaging, The First Affiliated Hospital of Chongqing Medical University, Chongqing, China

**Keywords:** hepatocellular carcinoma, radical resection, tumor location, propensity score matching, postoperative adjuvant therapy

## Abstract

**Aim:**

This study aims to explore the impact of the distance between the tumor and the main trunk or first branch of the portal vein on the prognosis of hepatocellular carcinoma (HCC) patients undergoing radical resection.

**Methods:**

This study retrospectively evaluated HCC patients who underwent radical resection between 1 January 2018 and 30 September 2024. Tumors were classified into two categories based on their location: central tumors and peripheral tumors. Central tumors were defined as those located within 2 cm of the main trunk or first branch of the portal vein, while the remaining tumors were classified as peripheral tumors. Recurrence-free survival (RFS) and overall survival (OS) were compared between the two groups. Univariate and multivariate COX analyses were conducted to identify factors associated with RFS and OS. Propensity score matching (PSM) was employed to eliminate intergroup differences for further validation.

**Results:**

A total of 667 HCC patients undergoing radical resection were initially enrolled. Through PSM, 247 patients were successfully matched in each comparative group. In the PSM cohort, the median RFS (mRFS) for patients with central tumors was 23.00 months (95% CI, 18.01–27.99 months), while the mRFS for those with peripheral tumors was 30.50 months (95% CI, 26.17–34.83 months) (p = 0.004). The median OS was 56.00 months (95% CI, 52.10–59.90 months) for central tumors and 72.00 months (95% CI, 67.37–76.63 months) for peripheral tumors (p = 0.043). Multivariate COX analysis confirmed that the distance of less than 2 cm between the tumor and the main trunk or first branch of the portal vein was an independent risk factor for RFS and OS in patients undergoing radical resection for HCC (HR: 1.744, p < 0.001; 1.728, p < 0.001, respectively).

**Conclusion:**

The distance between the tumor and the main portal vein trunk or first branch correlates with the prognosis of hepatocellular carcinoma patients undergoing radical resection.

## 1 Introduction

Primary liver cancer ranks sixth among the most common cancers worldwide and is the second leading cause of cancer-related deaths, particularly prevalent in developing countries with limited resources. Hepatocellular carcinoma (HCC) constitutes approximately 75%–85% of all primary liver cancer cases (Kocarnik et al., 2022; Bray et al., 2018). In China, with one of the largest populations worldwide and a high incidence of hepatitis B virus (HBV) infections, HCC ranks as the fifth most common cancer and the second leading cause of cancer-related deaths (Zheng et al., 2024). Radical treatment, such as ablation, radical liver resection (LR), and liver transplantation, is still the first choice for the treatment of HCC (Benson et al., 2021; Sun et al., 2022; European Association for the Study of the Liver, 2018). However, due to the inherent heterogeneity of HCC, there still remains a high recurrence rate even after radical resection. According to statistics, the recurrence rate within 5 years after radical resection remains as high as 50%–70%. Compared to patients without recurrence, the 5-year survival rate of patients with HCC recurrence is reduced by approximately 24% (Bruix et al., 2014; Kim et al., 2020; Tsilimigras et al., 2020a). Effectively preventing postoperative recurrence of HCC becomes crucial in improving the prognosis for patients with the disease.

It is widely believed that multiple tumors or satellite lesions, tumor diameter ≥5 cm, microvascular or macrovascular invasion, and poor differentiation are high-risk factors for recurrence after radical resection of HCC (Guo et al., 2023; Wakayama et al., 2017; Erstad and Tanabe, 2019; Lee et al., 2018). However, the impact of the distance between tumors and major blood vessels on tumor recurrence has not yet been studied. Bryant reported that tumor location was a predictive factor for local recurrence during short-term follow-up (Bryant et al., 2014). A study in Japan also indicated that treatment-naive HCCs in the peripheral zone had a longer local recurrence-free survival and progression-free survival following transarterial arterial chemoembolization (TACE) compared to those in the central zone. This suggests that the distance between tumors and major blood vessels may be related to tumor heterogeneity; the closer the distance, the higher the tumor activity and the faster the progression, leading to a worse prognosis (Asano et al., 2023). Other researchers have demonstrated that tumors located at the bifurcation of the portal vein, at the junction of the major hepatic vein, or within 1 cm of the inferior vena cava or the posterior aspect of the inferior vena cava have a recurrence rate as high as 90%, with a 5-year disease-free survival rate of only about 15%–30% (Cheng et al., 2012). It seems that the distance between tumors and major blood vessels may be related to the recurrence of HCC after radical resection, but there is currently limited research on this topic. Therefore, this study aims to evaluate the impact of tumor location on the recurrence of patients with HCC who have undergone radical LR.

## 2 Materials and methods

### 2.1 Patients

This study retrospectively evaluated patients with HCC who underwent liver resection (LR) at the Hepatobiliary Surgery Department of the First Affiliated Hospital of Chongqing Medical University from 1 January 2018 to 30 September 2024. The study was conducted in accordance with the Declaration of Helsinki, and was approved by the institutional ethics committee of the First Affiliated Hospital of Chongqing Medical University (2024–065–01). As this was a retrospective study, no additional patient consent was required.

**FIGURE 1:**
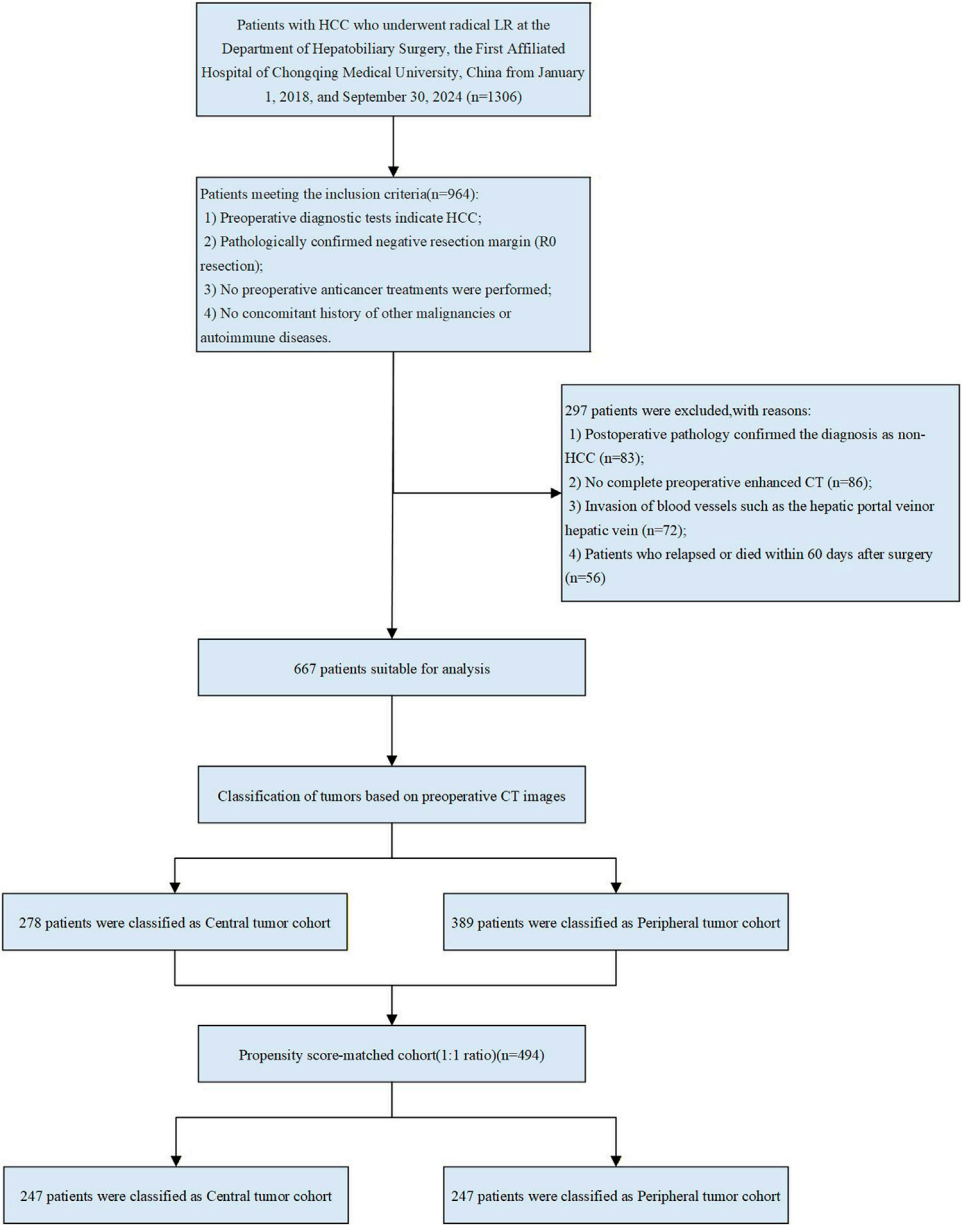
Flowchart of patient selection. HCC, hepatocellular carcinoma; LR, liver resection; CT, computed tomography.

Patients who met the following criteria were enrolled: 1. Postoperative pathological confirmation of HCC; 2. LR (negative margins confirmed by pathology); 3. LR as initial treatment; 4. No history of other malignancies or autoimmune diseases. Exclusion criteria: 1. R1 resection (postoperative pathology suggesting positive margins) or preoperative imaging suggesting extrahepatic metastases; 2. Preoperative or postoperative imaging confirmed the presence of portal vein and hepatic vein thrombosis; 3. Preoperative anticancer treatment; 4. Incomplete preoperative data; 5. Patients who relapsed or died within 60 days of surgery. The patient selection process is shown in Figure 1.

### 2.2 Radical LR and postoperative adjuvant therapy

All patients underwent routine preoperative examinations including ultrasound, enhanced computed tomography (CT) or magnetic resonance imaging (MRI) to assess tumor diameter, Barcelona Clinic Liver Cancer (BCLC) stage, resectable extent and residual liver volume. In addition, liver function was assessed using the Child-Pugh classification, and the degree of cirrhosis was evaluated using ICG R15 in all patients. The LR method contains non-anatomical resection and anatomical resection and the surgical technique used depends on the location and distribution of the tumor. Anatomical hepatectomy is the complete resection of the segment of liver with the tumor or the segment of liver limited by the branches of the portal vein of the tumor. Non-anatomical hepatectomy is the resection of the tumor and part of the non-tumor liver parenchyma (Zhou et al., 2001; Liu, 2024). Radical LR was defined as the complete removal of all detected tumors without involving any major branch of the portal or hepatic veins, without invasion of adjacent organs and without lymph node or distant metastasis, and tumor-free margins confirmed by histopathology (European Association for the Study of the Liver, 2018).

If patients are at high risk of hepatocellular carcinoma recurrence, postoperative adjuvant (PA) therapy should be considered, mainly including TACE, hepatic artery infusion chemotherapy (HAIC), immunotherapy, and targeted therapy. The TACE chemotherapy regimen typically combines chemotherapeutic agents such as oxaliplatin, irinotecan, fluorouracil, epirubicin, pirarubicin, and doxorubicin. These agents are either emulsified with iodized oil or loaded onto drug-eluting microspheres, then selectively administered via an arterial microcatheter into the tumor-feeding artery for targeted delivery. The drug regimen for HAIC mainly consists of the mFOLFOX regimen, which includes oxaliplatin, calcium folinate, and fluorouracil. The immunotherapy treatment regimen mainly includes Tislelizumab, Camrelizumab, and Atezolizumab. The targeted therapy treatment regimen mainly includes Sorafenib, Lenvatinib, Regorafenib, Apatinib, and Bevacizumab. Patients will decide whether to receive one or more adjuvant therapies and the frequency of treatment based on their own condition, financial status, or other social factors. Meanwhile, all patients with hepatitis B virus infection must receive lifelong antiviral treatment.

### 2.3 Tumor location

**FIGURE 2:**
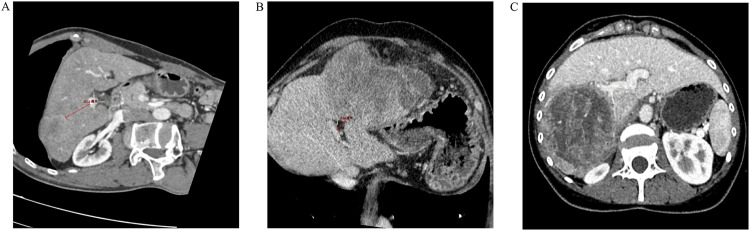
Distance of the tumor from the main trunk or first branch of the portal vein. **(A)** The distance from the tumor margin to the main trunk of the portal vein or the first branch is >2.0 cm (3.51 cm); **(B)** The distance from the tumor margin to the main trunk of the portal vein or the first branch is <2.0 cm (1.36 cm); **(C)** The tumor margin is in close contact with the right branch of the portal vein (but not invaded).

Preoperative enhanced CT was performed, using at least a 64-slice 128-layer spiral CT. Thin-slice images (1.00–1.25 mm) were obtained during the portal phase or venous phase (with clear portal vein enhancement). The main trunk of the portal vein, the right and left branches of the portal vein, and the second branches were identified on axial or multi-planar reconstruction (MPR). The shortest distance from the tumor margin to the main trunk or first branch of the portal vein was measured using MPR reconstruction (with distances touching the main trunk or first branch of the portal vein recorded as 0), accurate to the millimeter. Based on their location, tumors were classified as central or peripheral tumors. Tumors in the central zone meant that any portion of the tumors was present within 2 cm of the main trunk or first branch of the portal vein (central tumor), and Tumors outside this zone are classified as peripheral tumors (Figure 2). The measurement of the distance from the tumor to the main trunk or first branch was performed by two resident physicians, and if there was a significant discrepancy (≥0.3 cm), a physician with the title of associate chief physician or higher made the final decision.

### 2.4 Follow up and outcomes

All patients were followed up every 1–2 months for 6 months after discharge from the hospital and every 3–6 months thereafter. During the follow-up period, each patient received routine blood tests, liver function tests, AFP and abdominal ultrasound. If recurrence was suspected, enhanced CT or enhanced MRI was used to confirm the diagnosis. Recurrence was defined as any tumor nodule confirmed by two imaging studies or puncture biopsy. The primary endpoint of the study is recurrence-free survival (RFS), defined as the time from LR to the diagnosis of tumor recurrence, loss to follow-up, or the end of the follow-up period. The secondary endpoint is overall survival (OS), defined as the time from LR to death, loss to follow-up, or the end of the follow-up period. All patients are followed until 1 March 2025, loss to follow-up, or death.

### 2.5 Propensity score matching (PSM)

PSM analysis was conducted to minimize the uneven distribution of covariates between the two groups. The matching algorithm included age, gender, BCLC stage, presence or absence of hepatitis, presence or absence of cirrhosis, Alpha-fetoprotein (AFP) levels, albumin-bilirubin (ALBI) grade, Child-Pugh grade, neutrophil-to-lymphocyte ratio (NLR), platelet-lymphocyte ratio (PLR), alanine aminotransferase (ALT), total protein, γ-glutamyl transferase (GGT), number of tumors, tumor diameter, presence or absence of microvascular invasion (MVI), and postoperative adjuvant therapy regimen. A 1:1 nearest neighbor matching algorithm was used with a caliper width of 0.02. PSM was performed using SPSS 27.0 statistical software (IBM Corp, Armonk, NY, United States).

### 2.6 Statistical analysis

Statistical analyses were performed using SPSS version 27.0. The Shapiro-Wilk test was used to test the normality of continuous variables, and the independent samples t-test was used to detect continuous data that followed the normal distribution, expressed as the mean ± standard deviation. The Mann-Whitney U test was used to detect continuous data that were not normally distributed, expressed as median (interquartile range, IQR). Categorical data were detected using the chi-squared test, and expressed as numbers (n) and proportions (%). Univariate and multivariate analyses were performed in Cox risk models. Multivariate Cox regression analysis was performed using the Enter method, in which all candidate variables were entered simultaneously into the model and the significance of individual variables assessed by the Wald test. In addition, to verify the robustness of our findings, a forward-selection procedure was applied. Survival analyses were performed using the Kaplan–Meier method, and differences in the survival curves were analyzed using the log-rank test. K-M curves were plotted using R software (version 4.2.1 http://www.r-project.org). P-value < 0.05 was considered statistically significant.

## 3 Results

### 3.1 Baseline patient characteristics

After screening, a total of 667 eligible patients were included in this study. In the entire cohort, the age of the patients ranged from 56.01 ± 11.37 years, with 576 males (86.36%) and 91 females (13.64%). There were 278 patients (41.68%) with tumors located in the central region and 389 patients (58.32%) with tumors located in the peripheral region. Compared to tumors located in the peripheral region, tumors in the central region were larger (4.75 cm vs. 4.30 cm, p = 0.002), while no differences were observed in other baseline characteristics. Regarding surgical-related variables, patients with central tumors had longer surgical times (265 min vs. 230 min, p < 0.001) and experienced more intraoperative bleeding (300 mL vs. 200 mL, p < 0.001). However, no statistical differences were found between the two groups in terms of resection methods, MVI-positive/negative, tumor differentiation grading, or whether blood transfusions were administered.

In the PSM cohort, the imbalance between the two groups caused by differences in tumor size has been eliminated, and no significant differences were observed in the baseline characteristics of the two groups of patients. A total of 249 patients were matched in each group, with an overall patient age of 56.17 ± 11.42 years, including 430 males (87.04%) and 64 females (12.96%). The baseline information of patients in both the entire cohort and the PSM cohort is summarized in Tables 1, 2.

**TABLE 1:** Baseline characteristics of HCC patients between central tumor cohort and peripheral tumor cohort.

Characteristics		The entire cohort			The PSM cohort		
		Central tumor (n = 278)	Peripheral tumor (n = 389)	p	Central tumor (n = 247)	Peripheral tumor (n = 247)	p
Age, years		55.99 ± 11.09	56.03 ± 11.58	0.965	56.34 ± 10.99	56.00 ± 11.85	0.738
Age, n (%)	<56 years	139 (50.00)	188 (48.33)	0.670	119 (48.18)	119 (48.18)	1.000
	≥56 years	139 (50.00)	201 (51.67)		128 (51.82)	128 (51.82)	
Sex, n (%)	Male	238 (85.61)	337 (86.63)	0.706	215 (87.04)	215 (87.04)	1.000
	Female	40 (14.39)	52 (13.37)		32 (12.96)	32 (12.96)	
Hepatitis, n (%)	HBV	235 (84.53)	343 (88.17)	0.323	209 (84.62)	213 (86.24)	0.869
	HCV	3 (1.08)	5 (1.29)		3 (1.21)	3 (1.21)	
	No hepatitis	40 (14.39)	41 (10.54)		35 (14.17)	31 (12.55)	
Liver cirrhosis yes, n (%)		158 (56.83)	237 (60.93)	0.289	142 (57.49)	151 (61.13)	0.410
AFP, n (%)	<400 ng/mL	180 (64.75)	270 (69.41)	0.205	166 (67.21)	166 (67.21)	1.000
	≥400 ng/mL	98 (35.25)	119 (30.59)		81 (32.79)	81 (32.79)	
Tumor diameter, cm		4.75 (3.38, 7.00)	4.30 (2.90, 6.20)	0.002	4.60 (3.20, 6.50)	4.60 (3.10, 6.70)	0.966
Tumor diameter, n (%)	<5 cm	141 (50.72)	210 (53.98)	0.405	134 (54.25)	133 (53.85)	0.928
	≥5 cm	137 (49.28)	179 (46.02)		113 (45.75)	114 (46.15)	
Tumor number, n (%)	Solitary	215 (77.34)	299 (76.86)	0.886	200 (78.54)	200 (78.54)	1.000
	Multiple	63 (22.66)	90 (23.14)		47 (21.46)	47 (21.46)	
BCLC grade, n (%)	0+A	219 (78.78)	320 (82.26)	0.260	203 (82.19)	205 (83.00)	0.911
	B	59 (21.22)	69 (17.74)		44 (17.81)	42 (17.00)	
Child-Pugh grade, n (%)	A	270 (99.26)	378 (98.18)	0.240	245 (99.19)	245 (99.19)	1.000
	B	2 (0.74)	7 (1.82)		2 (0.81)	2 (0.81)	
ALBI score		−2.79 (−3.09, −2.48)	−2.79 (−3.09, −2.51)	0.729	−2.83 (−3.11, −2.50)	−2.77 (−3.08, −2.45)	0.491
ALBI grade, n (%)	1	179 (65.81)	264 (68.57)	0.457	166 (67.21)	159 (64.37)	0.507
	2	93 (34.19)	121 (31.43)		81 (32.79)	88 (35.63)	
Postoperative adjuvant treatment regimens, n (%)	NA	101 (36.33)	149 (38.30)	0.721	90 (36.44)	98 (39.68)	0.448
	PA-TACE	102 (36.69)	123 (31.62)		92 (37.24)	75 (30.36)	
	PA-HAIC	40 (14.39)	62 (15.94)		34 (13.77)	39 (15.79)	
	PA-targeted immunotherapy	35 (12.59)	55 (14.14)		31 (12.55)	35 (14.17)	

PSM, propensity score matching; HBV, hepatitis B virus; HCV, hepatitis C virus; AFP, alpha-fetoprotein; BCLC, barcelona clinic liver cancer; ALBI, albumin-bilirubin; ALBl = (log10 bilirubin * 0.66) + (albumin * −0.085); ALBI level 1, ≤ −2.60; ALBI level 2, -2.60 ∼ −1.39; PA, postoperative adjuvant; LR, liver resection; TACE, transcatheter arterial chemoembolization; HAIC, hepatic artery perfusion chemotherapy.

**TABLE 2:** Surgery-related variables during radical LR between central tumor cohort and peripheral tumor cohort.

Characteristics		The entire cohort			The PSM cohort		
		Central tumor (n = 278)	Peripheral tumor (n = 389)	p	Central tumor (n = 247)	Peripheral tumor (n = 247)	p
Hemoglobin, n (%)	<130 g/L	102 (37.36)	140 (36.36)	0.793	87 (35.22)	89 (36.03)	0.851
	≥130 g/L	171 (62.64)	245 (63.64)		160 (64.78)	158 (63.97)	
NLR, n (%)	<2.2	104 (38.24)	160 (41.67)	0.377	100 (40.49)	96 (38.87)	0.713
	≥2.2	168 (61.76)	224 (58.33)		147 (59.51)	151 (61.13)	
PLR, n (%)	<50	156 (57.35)	221 (57.55)	0.959	142 (57.49)	145 (58.70)	0.927
	≥50	116 (42.65)	163 (42.45)		105 (42.51)	102 (41.30)	
Total protein, n (%)	<70	128 (47.06)	189 (49.09)	0.608	117 (47.37)	124 (50.20)	0.529
	≥70	144 (52.94)	196 (50.91)		130 (52.63)	123 (49.80)	
ALT, n (%)	<35U/L	135 (49.63)	205 (53.25)	0.361	125 (50.61)	116 (46.96)	0.418
	≥35U/L	137 (50.37)	180 (46.75)		122 (49.39)	131 (53.04)	
GGT, n (%)	<60 U/L	141 (51.84)	214 (55.58)	0.310	134 (54.25)	128 (51.82)	0.589
	≥60U/L	131 (48.16)	171 (44.42)		113 (45.75)	119 (48.18)	
Resection pattern, n (%)	Anatomic	236 (84.89)	328 (84.32)	0.840	210 (85.02)	211 (85.43)	0.899
	Nonanatomic	42 (15.11)	61 (15.68)		37 (14.98)	36 (14.57)	
Hemorrhage, ml		300.00 (200.00, 500.00)	200.00 (100.00, 400.00)	<0.001	300.00 (200.00, 500.00)	200.00 (100.00, 400.00)	0.010
Operating time, minutes		265.00 (200.00, 325.00)	230.00 (180.00, 295.00)	<0.001	265.00 (200.00, 325.00	230.00 (180.00, 295.00)	<0.001
Blood transfusion, n (%)		33 (11.87)	47 (12.08)	0.934	30 (12.14)	30 (12.14)	1.000
Differentiation, n (%)	Low	57 (20.50)	73 (18.77)	0.576	44 (17.81)	38 (15.38)	0.468
	High and/or moderate	221 (79.50)	316 (81.23)		203 (82.19)	209 (84.62)	
MVI	Positive	182 (65.47)	237 (60.93)	0.231	157 (63.56)	149 (60.32)	0.458
	Negative	96 (34.53)	152 (39.07)		90 (36.44)	98 (39.68)	
Resection margin ≥ 1 cm, n (%)		278 (100.00)	389 (100.00)	1.000	247 (100.00)	247 (100.00)	1.000

PSM, propensity score matching; NLR, neutrophil-to-lymphocyte ratio; PLR, platelet-lymphocyte ratio; ALT, alanine aminotransferase; MVI, microvascular invasion; GGT, γ-Glutamyltransferase.

### 3.2 RFS

**FIGURE 3:**
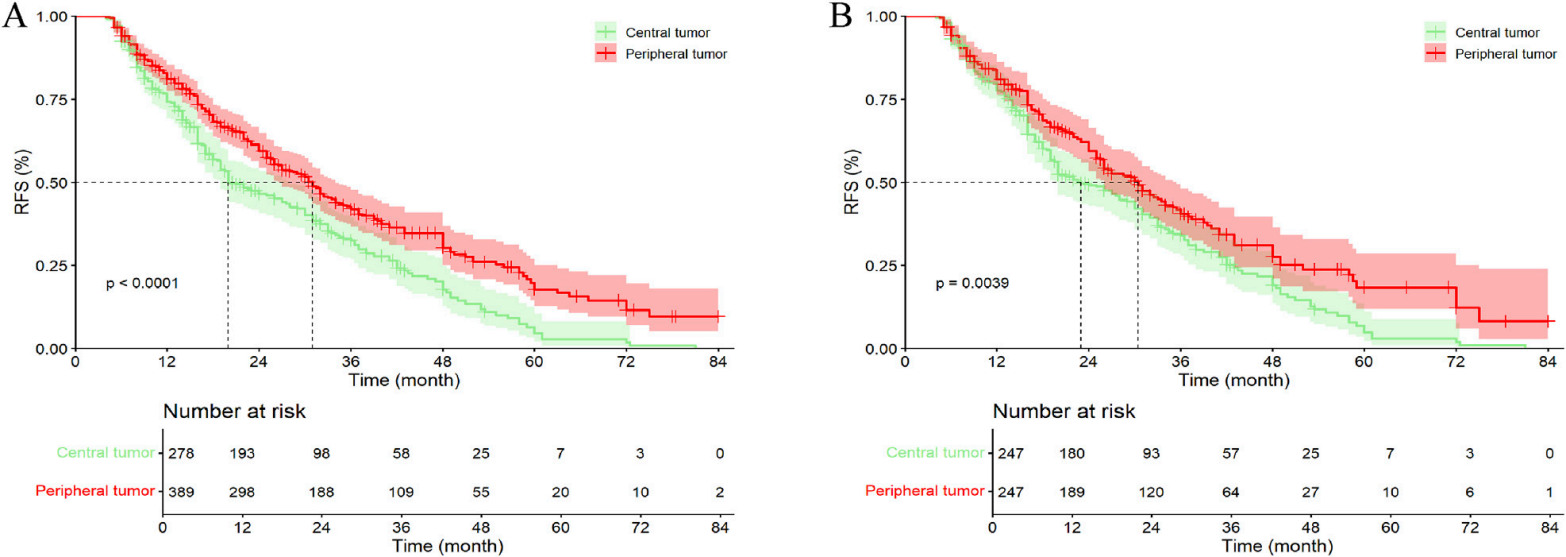
Kaplan–Meier analysis RFS of HCC patients between central tumor cohort and peripheral tumor cohort. **(A)** the entire cohort; **(B)** the PSM cohort; RFS, recurrence-free survival.

In the entire cohort, the median follow-up time for all patients was 41.00 months (95% CI, 36.96–45.06 months). Among them, 201 patients (72.30%) with central tumors experienced recurrence or were lost to follow-up, while 234 patients (60.15%) with peripheral tumors experienced recurrence or were lost to follow-up. The median RFS (mRFS) for patients with central tumors was 20.00 months (95% CI, 15.70–24.31), compared to 31.00 months (95% CI, 27.22–34.78) for those with peripheral tumors (p < 0.001). Furthermore, the 1-, 2-, 3-, 4-, 5-, and 6-year RFS rates for patients with central tumor were significantly lower than those for patients with peripheral tumor (Figure 3A; Supplementary Table S1A).

In the PSM cohort, the median follow-up time for all patients was 39.00 months (95% CI, 36.69–41.31 months). Among them, 179 patients (72.47%) with central tumors experienced recurrence or were lost to follow-up, while 145 patients (58.70%) with peripheral tumors experienced recurrence or were lost to follow-up. The mRFS for patients with central and peripheral tumors were 23.00 months (95% CI, 18.01–27.99 months) and 30.50 months (95% CI, 26.17–34.83 months), respectively (p = 0.004). Except for the 1- and 3-year RFS rates, patients with peripheral tumor exhibited higher RFS at all other time points compared to those with central tumor, with statistically significant differences (Figure 3B; Supplementary Table S1A). Additionally, in the Kaplan–Meier curves, after 72 months, the survival curves of both groups gradually converged, which may be due to the small number of patients remaining recurrence-free at 72 months, resulting in a gradual reduction of the difference between the groups.

### 3.3 OS

**FIGURE 4:**
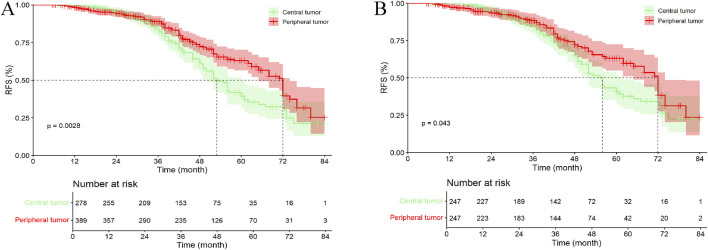
Kaplan–Meier analysis OS of HCC patients between central tumor and peripheral tumor. **(A)** the entire cohort; **(B)** the PSM cohort; OS, overall survival.

In the entire cohort, 96 patients (34.53%) with central tumors died or were lost to follow-up, while 104 patients (26.74%) with peripheral tumors died or were lost to follow-up. The median OS (mOS) for patients with central and peripheral tumors were 53.00 months (95% CI, 49.08–56.92 months) and 72.00 months (95% CI, 67.82–76.18 months), respectively (p = 0.003). Except for the 4- and 5-year OS rates, no statistically significant differences were observed between patients with central tumor and peripheral tumor (Figure 4A; Supplementary Table S1B).

In the PSM cohort, 84 patients (34.01%) with central tumors died or were lost to follow-up, while 65 patients (26.32%) with peripheral tumors died or were lost to follow-up. The mOS for patients with central and peripheral tumors were 56.00 months (95% CI, 52.10–59.90 months) and 72.00 months (95% CI, 67.37–76.63 months), respectively (p = 0.043). Similar to the entire cohort, except for the 5-year OS rate, no statistically significant differences were observed between patients with central tumor and peripheral tumor (Figure 4B; Supplementary Table S1B).

### 3.4 COX regression analysis

Univariate and multivariate COX regression analyses were conducted for both the entire cohort and the PSM cohort (Specific details are given in Tables 3, 4; Supplementary Table S2). In the entire cohort, the following factors were identified as independent risk factors for RFS: tumor location <2 cm (1.900, 95% CI 1.559, 2.317), tumor diameter ≥5 cm (4.705, 95% CI 3.707, 5.917), multiple tumors (2.320, 95% CI 1.353, 3.979), poor differentiation (2.384, 95% CI 1.868, 3.041), and MVI (1.555, 95% CI 1.241, 1.948). Postoperative adjuvant therapy (p < 0.001) was identified as an independent protective factor for RFS. Additionally, tumor location <2 cm (1.731, 95% CI 1.303, 2.299), tumor diameter ≥5 cm (3.645, 95% CI 2.621, 5.069), and MVI (1.985, 95% CI 1.395, 2.824) were identified as independent risk factors for OS. The same conclusions were reached in the PSM cohort. In addition, a Cox regression analysis using the forward-selection variable entry method was performed for verification, with details provided in Supplementary Table S3.

**TABLE 3:** Multivariate analysis of RFS in the entire cohort and the PSM cohort.

Characteristics		The entire cohort		The PSM cohort	
		HR (95% CI)	p	HR (95% CI)	p
Tumor location, cm (≤2 vs. > 2)		1.900 (1.559, 2.317)	<0.001	1.744 (1.389, 2.190)	<0.001
Tumor diameter, cm (≥5 vs. < 5)		4.705 (3.707, 5.917)	<0.001	5.050 (3.813, 6.687)	<0.001
Tumor number (multiple vs. single)		2.320 (1.353, 3.979)	0.002	3.722 (1.614, 8.585)	0.002
BCLC (B vs. 0+A)		0.879 (0.500, 1.545)	0.655	0.539 (0.227, 1.281)	0.162
PA-treatment regimens	LR	Reference		Reference	
	PA-TACE	0.489 (0.388, 0.615)	<0.001	0.505 (0.388, 0.658)	<0.001
	PA-HAIC	0.386 (0.274, 0.545)	<0.001	0.363 (0.240, 0.548)	<0.001
	PA-targeted immunotherapy	0.514 (0.359, 0.737)	<0.001	0.544 (0.365, 0.811)	0.003
Differentiation (low vs. high and/or moderate)		2.384 (1.868, 3.041)	<0.001	2.539 (1.889, 3.413)	<0.001
MVI (positive vs. negative)		1.555 (1.241, 1.948)	<0.001	1.478 (1.137, 1.921)	0.004
NLR (≥2.2 vs. < 2.2)		1.061 (0.867, 1.298)	0.567	-	-

PSM, propensity score matching; BCLC, barcelona clinic liver cancer; PA, postoperative adjuvant; LR, liver resection; TACE, transcatheter arterial chemoembolization; HAIC, hepatic artery perfusion chemotherapy; MVI, microvascular invasion; NLR, neutrophil-to-lymphocyte ratio.

**TABLE 4:** Multivariate analysis of OS in the entire cohort and the PSM cohort.

Characteristics	The entire cohort		The PSM cohort	
	HR (95% CI)	p	HR (95% CI)	p
Tumor location,cm (≤2 vs. > 2)	1.731 (1.303, 2.299)	<0.001	1.728 (1.240, 2.409)	<0.001
Tumor diameter, cm (≥5 vs. < 5)	3.645 (2.621, 5.069)	<0.001	3.706 (2.533, 5.423)	<0.001
Tumor number (multiple vs. single)	1.765 (0.706, 4.410)	0.160	-	-
Differentiation (low vs. high and/or moderate)	1.242 (0.896, 1.721)	0.194	0.974 (0.647, 1.467)	0.902
MVI (positive vs. negative)	1.985 (1.395, 2.824)	<0.001	2.023 (1.358, 3.013)	<0.001
BCLC (B vs. 0+A)	0.803 (0.310, 2.081)	0.651	-	-

PSM, propensity score matching; MVI, microvascular invasion; BCLC, barcelona clinic liver cancer.

### 3.5 All exploratory subgroup analysis

**FIGURE 5:**
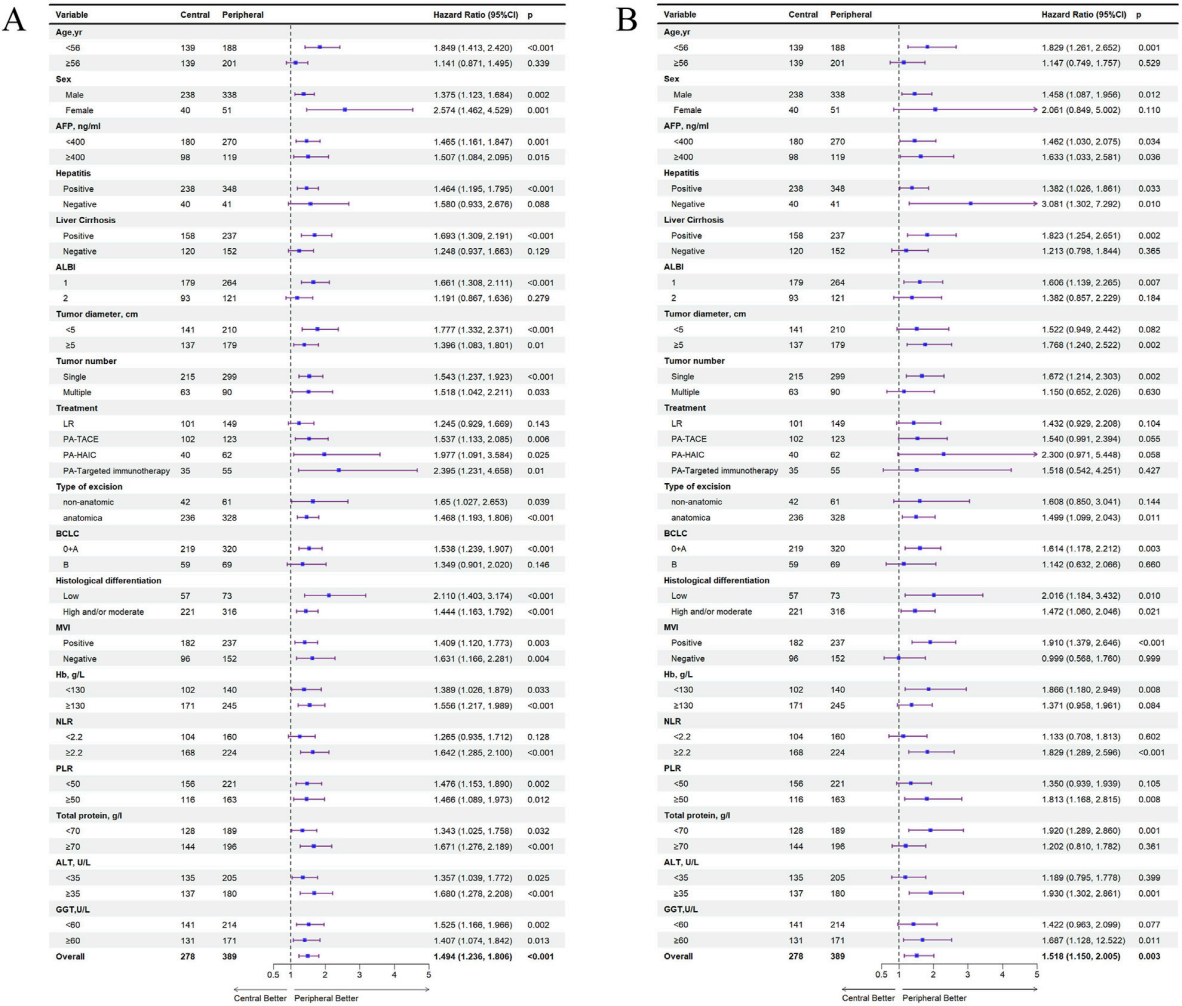
All exploratory subgroup analysis for patients between central tumor and peripheral tumor in the entire cohort. **(A)** All exploratory subgroup analysis about RFS; **(B)** All exploratory subgroup analysis about OS; AFP, alpha-fetoprotein; ALBI, albumin-bilirubin; ALBI grade 1, ≤ −2.60; ALBI grade 2, −2.60 ∼ −1.39; PA, postoperative adjuvant; LR, liver resection; TACE, transcatheter arterial chemoembolization; HAIC, hepatic artery perfusion chemotherapy; BCLC, barcelona clinic liver cancer; NLR, neutrophil-to-lymphocyte ratio, PLR, platelet-to-lymphocyte ratio, ALT, alanine aminotransferase, MVI, microvascular invasion; GGT, γ-Glutamyltransferase.

In the entire cohort, significant differences between central tumor cohort and peripheral tumor cohort regarding the recurrence of HCC were observed in populations aged <56 years, with concomitant hepatitis or cirrhosis, ALBI grade 1, PA-TACE, PA-HAIC, PA-Targeted immunotherapy, and BCLC stages 0+A. Also, significant differences in OS were observed aged <56 years, male patients, those with concomitant cirrhosis, ALBI grade 1, tumors ≥5 cm, single tumors, anatomical resection, BCLC stages 0+A, MVI positive, NLR ≥2.2, and PLR ≥50. Specific details have been shown in Figure 5. In the PSM cohort, the same conclusion was reached (Supplementary Figure S1).

### 3.6 Comparative efficacy of identical postoperative adjuvant therapies between central and peripheral tumor groups

**FIGURE 6:**
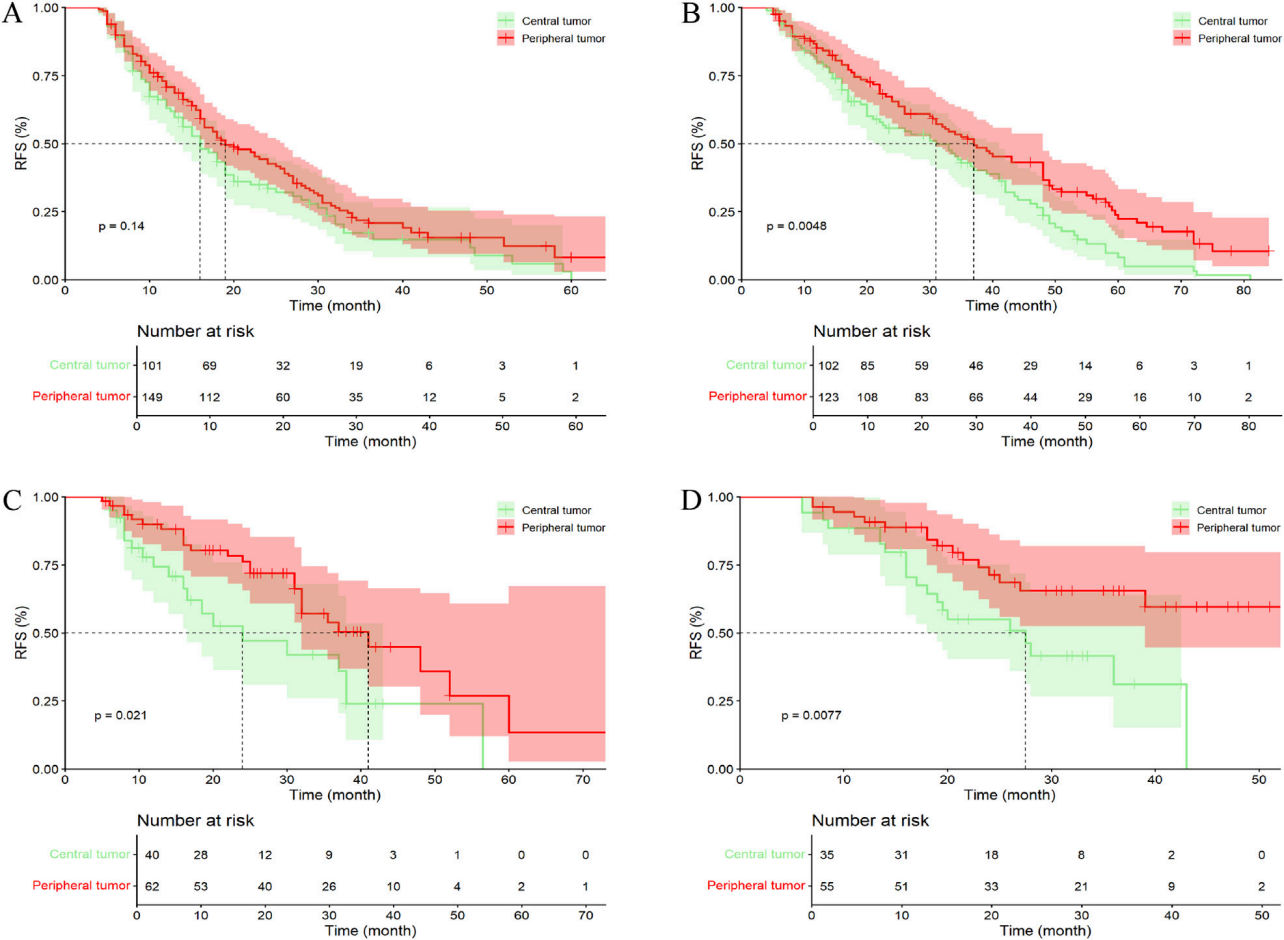
Comparative efficacy of identical postoperative adjuvant therapies between central tumor and peripheral tumor in the entire cohort. **(A)** Patients Undergoing LR; **(B)** Patients Undergoing PA-TACE; **(C)** Patients Undergoing PA-HAIC; **(D)** Patients Undergoing PA-Targeted immunotherapy.

In the entire cohort, 101 patients (36.33%) with central tumor underwent LR alone, 102 patients (36.90%) received PA-TACE, 40 patients (14.39%) received PA-HAIC, and 35 patients (12.59%) received postoperative targeted immunotherapy. Among patients with peripheral tumor, 149 patients (38.30%) underwent LR alone, 123 patients (31.62%) received PA-TACE, 62 patients (15.59%) received PA-HAIC, and 55 patients (14.14%) received PA-Targeted immunotherapy. The results showed that, among patients who underwent surgery alone, those with peripheral tumor had a better mRFS than those with central tumor; however, the difference was not statistically significant (p = 0.136). In contrast, patients who received PA-TACE, PA-HAIC, or PA-targeted immunotherapy had significantly prolonged mRFS compared with those who did not receive any adjuvant therapy (p = 0.005, 0.021, and 0.008, respectively) (Figure 6; Supplementary Table S4).

**FIGURE 7:**
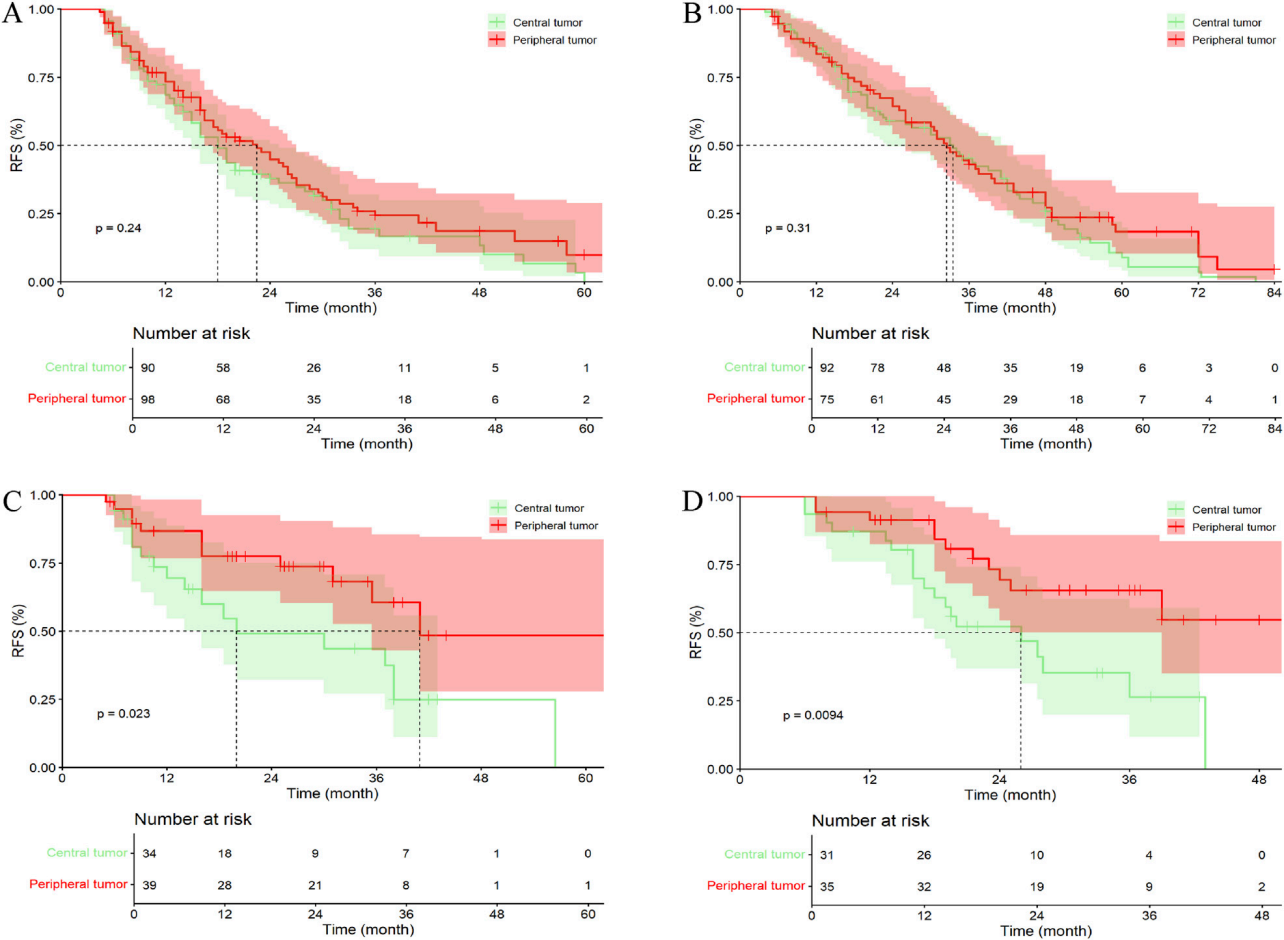
Comparative efficacy of identical postoperative adjuvant therapies between central tumor and peripheral tumor in the PSM cohort. **(A)** Patients Undergoing LR; **(B)** Patients Undergoing PA-TACE; **(C)** Patients Undergoing PA-HAIC; **(D)** Patients Undergoing PA-Targeted immunotherapy.

In the PSM cohort, 90 patients (36.44%) with central tumors underwent surgery alone, 92 patients (37.24%) received PA-TACE, 34 patients (13.77%) received PA-HAIC, and 31 patients (12.55%) received PA-Targeted immunotherapy, whereas in the peripheral tumor group, 98 patients (39.68%) underwent surgery alone, 75 patients (30.36%) received PA-TACE, 39 patients (15.79%) received PA-HAIC, and 35 patients (14.17%) received PA-Targeted immunotherapy targeted immunotherapy. Unlike the results observed in the entire cohort, no significant difference in mRFS was found between central and peripheral tumor groups among patients who received PA-TACE (p = 0.310) (Figure 7; Supplementary Table S4).

### 3.7 Efficacy assessment of different postoperative adjuvant therapies in central and peripheral tumor groups

**FIGURE 8:**
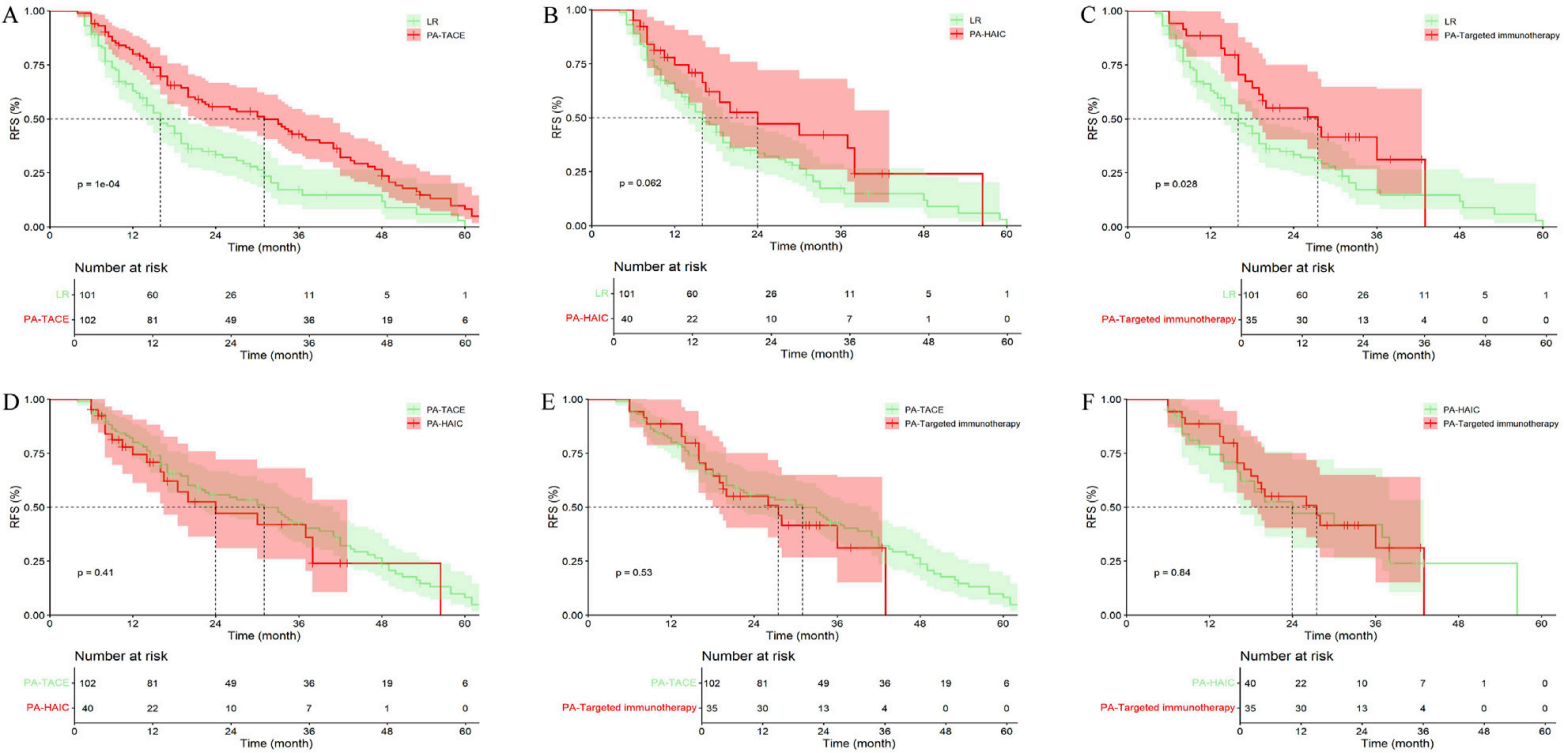
Comparison of RFS in central tumor patients receiving different postoperative adjuvant therapies. **(A)** LR vs. PA-TACE; **(B)** LR vs. PA-HAIC; **(C)** LR vs. PA-Targeted immunotherapy; **(D)** PA-TACE vs. PA-HAIC; **(E)** PA-TACE vs. PA-Targeted immunotherapy; **(F)** PA-HAIC vs. PA-Targeted immunotherapy; PA, postoperative adjuvant; LR, liver resection; TACE, transcatheter arterial chemoembolization; HAIC, hepatic artery perfusion chemotherapy.

In the entire cohort, 101 patients (36.33%) with central tumor did not receive any treatment, 102 patients (36.69%) received PA-TACE, 40 patients (14.39%) received PA-HAIC, and 35 patients (12.59%) received PA-Targeted immunotherapy. It was ultimately found that patients receiving PA-TACE and PA-Targeted immunotherapy had significantly longer mRFS compared to those who did not receive adjuvant treatment. Although patients receiving PA-HAIC had better mRFS than those who did not receive adjuvant treatment, the difference was not statistically significant. However, there were no significant differences in mRFS among the PA-TACE, PA-HAIC, and PA-Targeted immunotherapy (Figure 8; Supplementary Table S5).

**FIGURE 9:**
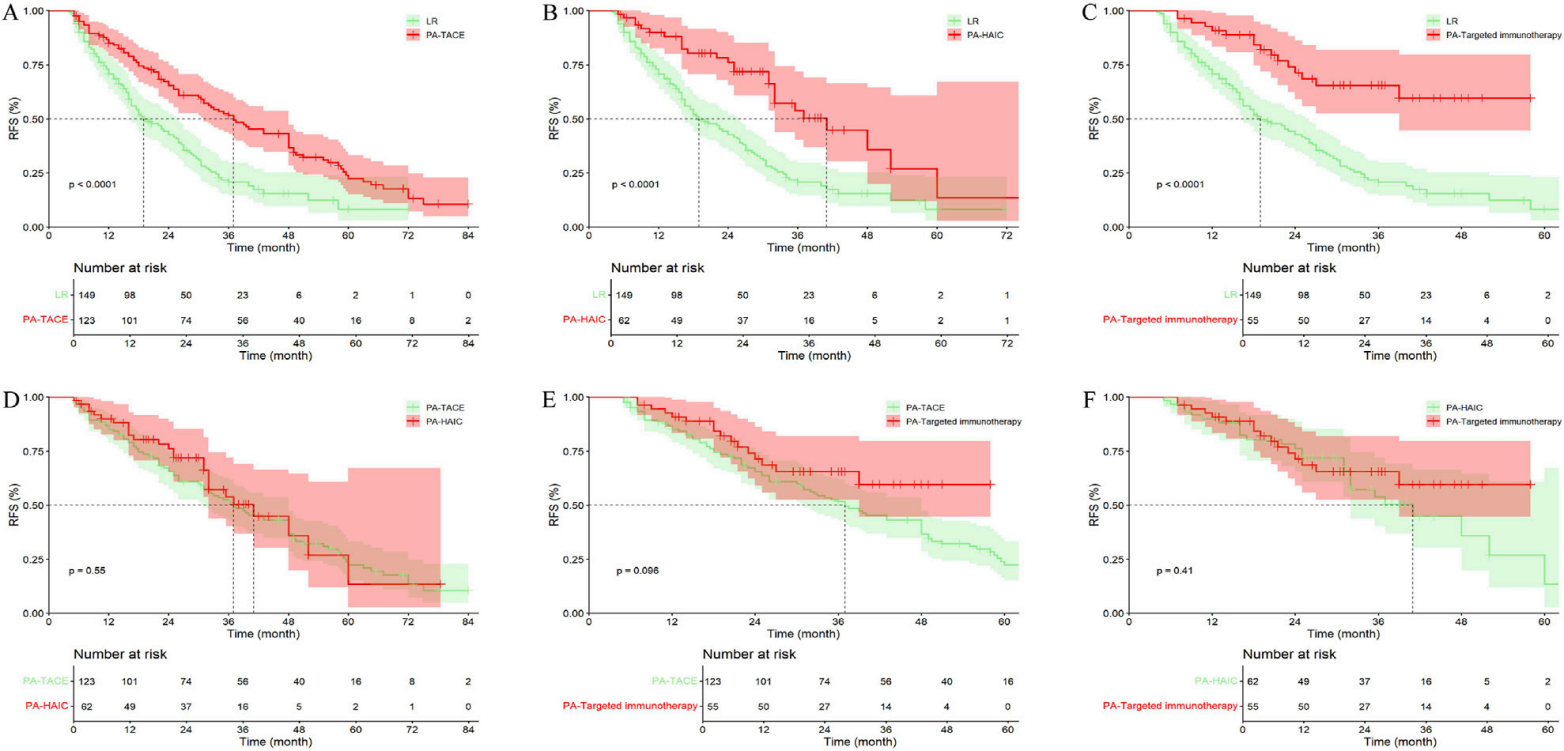
Comparison of RFS in peripheral tumor receiving different postoperative adjuvant therapies. **(A)** LR vs. PA-TACE; **(B)** LR vs. PA-HAIC; **(C)** LR vs. PA-Targeted immunotherapy; **(D)** PA-TACE vs. PA-HAIC; **(E)** PA-TACE vs. PA-Targeted immunotherapy; **(F)** PA-HAIC vs. PA-Targeted immunotherapy; PA, postoperative adjuvant; LR, liver resection; TACE, transcatheter arterial chemoembolization; HAIC, hepatic artery perfusion chemotherapy.

Among patients with peripheral tumor, 149 patients (38.30%) did not receive any treatment, 123 patients (31.62%) received PA-TACE, 62 patients (15.94%) received PA-HAIC, and 55 patients (14.14%) received PA-Targeted immunotherapy. The overall results were similar to those observed in patients with central tumors (Figure 9; Supplementary Table S5).

### 3.8 Recurrence patterns

In the entire cohort, a total of 201 patients (72.30%) with central tumors experienced recurrence, while 234 patients (60.15%) with peripheral tumors experienced recurrence. In the PSM cohort, 179 patients (72.47%) with central tumors experienced recurrence, and 145 patients (58.70%) with peripheral tumors experienced recurrence. Using 2 years as a cutoff point, recurrences were categorized into early recurrence and late recurrence. In both the entire cohort and the PSM cohort, there were no statistically significant differences in recurrence patterns and recurrence sites between the two groups (Table 5).

**TABLE 5:** Comparison of recurrence patterns and recurrence sites between patients with central tumor and peripheral tumor.

Characteristics		The entire cohort			The PSM cohort		
		Central tumor (n = 201) (%)	Peripheral tumor (n = 234) (%)	p	Central tumor (n = 179) (%)	Peripheral tumor (n = 145) (%)	p
Recurrence pattern	Early recurrence	134 (66.77)	143 (61.11)	0.230	113 (63.13)	90	0.845
	Late recurrence	67 (33.33)	91 (38.89)		66 (36.87)	55	
Recurrence site	Intrahepatic recurrence	130 (64.68)	163 (69.66)	0.577	117 (65.37)	105	0.374
	Vascular invasion	39 (19.40)	35 (14.96)		35 (19.55)	24	
	Extrahepatic metastasis	32 (15.92)	36 (15.38)		27 (15.08)	16	

PSM, propensity score matching.

### 3.9 Complications and safety

In terms of safety, all patients underwent radical LR, and some patients received PA-TACE, PA-HAIC, and PA-Targeted immunotherapy. Both in the entire cohort and the PSM cohort, patients with central tumors had longer surgical times and more intraoperative bleeding compared to those with peripheral tumors (Table 1). This is primarily due to the proximity of central tumors to major blood vessels, which increases the risk of bleeding and complicates the surgical procedure, resulting in longer operation times. Additionally, all patients experienced only mild complications during surgery and postoperative adjuvant treatment, which improved with symptomatic treatment, and no severe adverse reactions were observed. There were also no perioperative deaths.

## 4 Discussion

Our study ultimately demonstrates that, among HCC patients who underwent radical resection those with central tumor had poorer RFS and OS compared with patients with peripheral tumor, suggesting that the distance between the tumor and the portal vein may also be an important factor in HCC recurrence. However, in both the entire cohort and the PSM cohort, patients who underwent LR alone showed superior RFS for peripheral tumor compared to central tumor, though this difference was not statistically significant. This may be attributed to the relatively short RFS in both groups, resulting in an indistinguishable difference.

HCC is a highly aggressive tumor, and its main pathological dissemination route is through the portal vein territory. This mode of spread is also one of the significant reasons for intrahepatic metastasis and local recurrence after surgery. The closer the tumor is to the portal vein, the greater the likelihood of tumor dissemination (Shindoh et al., 2016; Viganò et al., 2018). A retrospective study included 2,799 patients and ultimately demonstrated that, compared to tumors located in the right lobe of the liver, patients with tumors in the left lobe had poorer OS and RFS after undergoing radical resection (OS: 66.00 vs. 72.00 months, P = 0.001; RFS: 28.00 vs. 51.00 months, P < 0.001, respectively) (Tang et al., 2023). In addition to the authors’ speculations regarding differences in genetic background, immunopathology, and recurrence patterns between the left and right lobes of the liver, we hypothesize that the proximity of tumors in the left lobe to the main trunk or first branch of the portal vein may also be a contributing factor (Tang et al., 2023; Nevola et al., 2023; Hikspoors et al., 2017). In traditional concepts, central zone tumors are defined as tumor masses located in the Couinaud segments IV, V, and VIII of the liver (Wu et al., 1999). Several studies have shown that tumors located at the segmental boundary and in the central liver (Couinaud segments I or IV) are independent risk factors for prognosis in patients with unresectable HCC undergoing TACE treatment (Chen et al., 2021; Vesselle et al., 2016; Park et al., 2007). Bryant et al. demonstrated that, among patients with HCC undergoing initial TACE treatment, complete or >90% tumor necrosis was achieved in only 23% of centrally located tumors (defined as being within 4 cm of the portal vein bifurcation). In comparison, peripherally located HCC lesions were significantly more likely to achieve a favorable response (OR 3.60, 95% CI 1.27–11.87) (Bryant et al., 2014). Another study further explored the impact of the distance between tumors and the portal vein, hepatic vein, and bile ducts on tumor prognosis. A retrospective study included 135 patients with BCLC stage 0 or A HCC who underwent conventional TACE (cTACE), ultimately demonstrating that central zone tumors (defined as tumors located within 0.5 cm of the first or second branch of the portal vein) are an important factor associated with poor prognosis in early HCC patients after cTACE (Progression-free survival: HR, 1.664, 95% CI 1.038–2.667; OS: HR, 1.890, 95% CI 1.021–3.497) (Lee et al., 2022). There are also numerous studies on the impact of tumor location on the efficacy of radiofrequency ablation (RFA). A retrospective comparative study indicated that in patients with HCC with a diameter of ≤3 cm undergoing RFA, the treatment outcomes for vascular-adjacent HCC were comparable to those for non-vascular-adjacent HCC (P = 0.689 for disease-free survival and 0.267 for OS) (Kang et al., 2014). However, a retrospective study by Shoujin Cao et al. compared the prognostic impact of perivascular HCC (tumor margin within 5 mm of the main portal vein, including the first to third branches) versus non-perivascular tumors undergoing RFA. The study demonstrated that the perivascular group had significantly higher local tumor progression rates at 1, 3, and 5 years compared with the non-perivascular group (15.70%, 33.70%, and 46.90% vs. 6.00%, 15.70%, and 28.70%, P = 0.0067). Additionally, OS at 1, 3, and 5 years was significantly lower in the perivascular group than in the non-perivascular group (81.30%, 65.10%, and 42.90% vs. 99.30%, 90.40%, and 78.10%, P < 0.0001) (Cao et al., 2022). Several other studies have shown that a distance of ≤5 mm between the tumor and the first or second branches of the portal vein or hepatic vein is an important risk factor for intrahepatic recurrence, local tumor progression, and extrahepatic metastasis in patients with single nodular HCC ≤5 cm undergoing RFA (Lee et al., 2021; Kang et al., 2017; Huang et al., 2021). Changcheng Tao also proposed another classification method: HCC located within <1 cm of the hepatic vein, portal vein, biliary system, or inferior vena cava, which is typically confirmed by preoperative imaging, intraoperative visual inspection, or postoperative pathological results. This classification indicates that the tumors are usually located in Couinaud segments IV, V, and VIII, or at the junction of the central portion, similar to the previous classification but with a greater emphasis on the relationship with the vascular structures (Tao et al., 2024). In the surgical treatment of HCC, it is essential to ensure sufficient margins to completely remove micro-metastases of the tumor. Studies have shown that micro-metastases typically occur within 1 cm of the resection margin, and patients with a resection margin of <1 cm have higher recurrence rates and poorer survival outcomes compared to those with a margin of ≥1 cm (Liu et al., 2020; Zhou et al., 2007; Zhong et al., 2017; Tsilimigras et al., 2020b). In radical resection, a surgical margin of ≥1 cm is generally recommended (Zhou et al., 2023). Therefore, when exploring the impact of tumor location on the prognosis after radical resection of HCC, we believe that using a cutoff value of 1 cm for the distance between the tumor and the main trunk or first branch of the portal vein is not appropriate. After conducting a statistical analysis of the imaging data of the patients included in this study, we consider that a cutoff value of 2 cm for the distance between the tumor and the main trunk or first branch of the portal vein is relatively suitable.

TACE reduces intratumoral blood flow by depositing lipiodol within the tumour vasculature, thereby depriving the tumour of oxygen and nutrients. This process also prolongs local drug retention and enhances chemotherapeutic exposure, ultimately facilitating the eradication of residual tumour cells (Chen et al., 2013; Gaba et al., 2018). HAIC increases the exposure time of chemotherapeutic agents to the tumor through continuous infusion. Moreover, sustained high-flow infusion raises interstitial pressure gradients, improving intratumoral drug penetration and distribution, and thereby more effectively eradicating residual intrahepatic or circulating micro-metastases (Leal and Kingham, 2015; Liang et al., 2013; Ganeshan et al., 2008). Tyrosine kinase inhibitors target vascular endothelial growth factor receptors and have an overall inhibitory effect on tumor angiogenesis, which may help eliminate residual micro-metastases and prevent postoperative recurrence and metastasis of HCC (Qin et al., 2019). Immunotherapy, through anti-programmed cell death receptor 1 antibodies, modulates the immune microenvironment and induces T lymphocyte expansion, which is beneficial for eliminating small metastases in the liver, thereby achieving the effect of preventing tumor recurrence (Versluis et al., 2020). In our study, among both central tumor and peripheral tumor, patients receiving PA-TACE, PA-HAIC, or PA-targeted immunotherapy exhibited better RFS than those who underwent LR alone; however, no significant differences in efficacy were observed among the three adjuvant modalities. Given that residual HCC cells tend to disseminate along portal-venous branches, patients with peripheral tumor who received PA-HAIC or PA-targeted immunotherapy experienced significantly better RFS than those with central tumor. However, in the PSM cohort, no significant difference was observed between central tumor and peripheral tumor among patients treated with PA-TACE. This may reflect biological effects of transarterial chemoembolisation, which can upregulate HIF-1α, VEGF and regulators of epithelial–mesenchymal transition, thereby exacerbating hypoxia and reinforcing immunosuppression within the tumour microenvironment (Qu et al., 2015; Ader et al., 2008; Fang et al., 2013; Xu et al., 2019; Lin et al., 2021). Moreover, the greater the distance of a tumor from the portal vein and hepatic artery, the more severe the hypoxic insult induced by embolisation, which may promote recurrence (Miki et al., 2017). We therefore hypothesise that these mechanisms account for the lack of a significant difference between peripheral and central tumors following. Moreover, owing to the relatively small number of patients in our cohort who received postoperative PA-HAIC or PA-targeted immunotherapy, further studies are warranted to validate these findings.

This study still has some limitations. First, it is a single-center retrospective study, and the number of patients included is still not sufficient, especially for those who received PA-HAIC and PA-targeted immunotherapy, which may lead to significant bias and affect the relevance of the study results. Second, the follow-up period of this study needs to be extended, as the majority of patients have not reached the endpoint of survival, and the proportion of lost to follow-up patients is relatively high, which significantly impacts the comparison of OS between the two groups. Finally, most of the patients included in this study have hepatitis B infection. However, in Western countries, the main risk factor for hepatocellular carcinoma is fatty liver disease associated with metabolic dysfunction. The effects of these two conditions on the liver tumor microenvironment vary, potentially resulting in different final outcomes.

## 5 Conclusion

The distance of the tumor from the main portal vein trunk or first branch correlates with the prognosis of hepatocellular carcinoma patients undergoing radical resection.

## Data Availability

The original contributions presented in the study are included in the article/Supplementary Material, further inquiries can be directed to the corresponding author.
