# Supplementary material for: The therapeutic efficacy of radical resection for hepatocellular carcinoma varies markedly by tumor location: a retrospective real-world study
Source: Front Pharmacol. 2025 Dec 3;16:1674998. doi: 10.3389/fphar.2025.1674998 (PMC12708258; doi:10.3389/fphar.2025.1674998)
Supplement: Supplementary file 1 [file Supplementaryfile1.docx]

The therapeutic efficacy of radical resection for hepatocellular carcinoma varied markedly with tumor location, based on real-world data from a retrospective single-centre analysis

Xu Feng^1,2+^, Yi-Qiu Wei^3+^, Jia-Rui Liu^1,2^, Zheng-Rong Shi^2^, Kai Chen^2^, Yong-Shuang Lv^1^

1. Department of Hepatobiliary Surgery, The Affiliated Yongchuan Hospital of Chongqing Medical University, (China), Chongqing

2. Department of Hepatobiliary Surgery, The First Affiliated Hospital of Chongqing Medical University, (China), Chongqing

3. Department of Imaging, The First Affiliated Hospital of Chongqing Medical University, (China), Chongqing

Corresponding author: Yong-Shuang Lv: [lyshkill@qq.com](mailto:lyshkill@qq.com).

^+^The authors Xu Feng and Yi-Qiu Wei contributed equally to this work.

Supplementary table 1.A Comparison of RFS in HCC patients receiving different postoperative adjuvant therapy

| Characteristics | | mRFS (months) | 1-year RFS rate (%) | 2-year RFS rate (%) | 3-year RFS rate (%) | 4-year RFS rate (%) | 5-year RFS rate (%) | 6-year RFS rate (%) |
| --- | --- | --- | --- | --- | --- | --- | --- | --- |
| the Entire Cohort | Central tumor | 20.00 (15.70, 24.31) | 74.30 (69.01, 79.59) | 46.60 (40.33, 52.87) | 32.50 (26.23, 38.77) | 17.80 (11.92, 23.68) | 4.60 (0, 8.32) | 1.80 (0, 4.35) |
|  | Peripheral tumor | 31.00 (27.22, 34.78) | 81.20 (77.28, 85.12) | 59.40 (54.11, 64.69) | 41.90 (36.41, 47.39) | 30.30 (24.42, 36.18) | 17.80 (11.72, 23.88) | 11.60 (5.52, 17.68) |
| the PSM Cohort | Central tumor | 23.00 (18.01, 27.99) | 77.80 (72.51, 83.09) | 49.20 (42.54, 55.86) | 34.00 (27.34, 40.66) | 19.10 (12.83, 25.37) | 4.90 (0.78, 9.02) | 2.00 (0, 4.74) |
|  | Peripheral tumor | 30.50 (26.17, 34.83) | 81.10 (72.51, 83.09) | 59.50 (53.03, 65.97) | 40.50 (33.44, 47.56) | 27.70 (20.06, 35.34) | 18.40 (10.36, 26.44), | 12.30 (3.48, 21.12) |
| Comparison | | p value | | | | | | |
| the Entire Cohort | | <0.001 | 0.036 | 0.001 | 0.012 | <0.001 | <0.001 | <0.001 |
| the PSM Cohort | | 0.004 | 0.374 | 0.024 | 0.136 | 0.025 | <0.001 | <0.001 |

mRFS, median recurrence-free survival; PSM, propensity score matching.

Supplementary table 1.B Comparison of OS in HCC patients receiving different postoperative adjuvant therapy

| Characteristics | | mOS (months) | 1-year OS rate (%) | 2-year OS rate (%) | 3-year OS rate (%) | 4-year OS rate (%) | 5-year OS rate (%) | 6-year OS rate (%) |
| --- | --- | --- | --- | --- | --- | --- | --- | --- |
| the Entire Cohort | Central tumor | 53.00 (49.08. 56.92) | 99.60 (99.82, 100.00） | 95.70 (93.15, 98.25) | 83.60 (78.50, 88.70) | 61.50 (53.66, 69.34) | 39.40 (30.38, 48.42) | 29.80 (20.00, 39.60) |
|  | Peripheral tumor | 72.00 (67.82, 76.18) | 98.10 (96.73, 99.47) | 94.20 (91.65, 96.75) | 87.20 (83.48, 90.92) | 72.50 (66.62, 78.38) | 61.10 (53.85, 68.35) | 39.60 (29.41, 49.79) |
| the PSM Cohort | Central tumor | 56.00 (52.10, 59.90) | 99.10 (97.73, 100.00) | 95.70 (92.96, 98.44) | 84.00 (78.71, 89.29) | 65.20 (57.36, 73.04) | 40.70 (31.10, 50.30) | 31.70 (21.31, 42.09) |
|  | Peripheral tumor | 72.00 (67.37, 76.63) | 97.40 (95.44, 99.36) | 93.60 (90.46, 96.74) | 87.10 (82.40, 91.80) | 72.30 (64.85, 79.75) | 59.80 (50.39, 69.21) | 38.40 (25.46, 51.34) |
| Comparison | | p value | | | | | | |
| the Entire Cohort | | 0.003 | 0.057 | 0.240 | 0.276 | 0.049 | <0.001 | 0.077 |
| the PSM Cohort | | 0.043 | 0.143 | 0.322 | 0.308 | 0.080 | <0.001 | 0.109 |

mOS, median overall survival; PSM, propensity score matching

Supplementary table 2.A Univariate COX Regression Analysis of RFS in the Entire Cohort and the PSM Cohort

| Characteristics | | the Entire cohort | | the PSM cohort | |
| --- | --- | --- | --- | --- | --- |
|  |  | HR (95% CI) | p | HR (95% CI) | p |
| Tumor location, cm (≤2 vs >2) | | 1.494 (1.236, 1.806) | <0.001 | 1.373 (1.103, 1.710) | 0.005 |
| Age, yr（≥56 vs <56） | | 1.133 (0.937, 1.371) | 0.198 | 1.057 (0.848, 1.317) | 0.622 |
| Sex（Male vs Female） | | 1.293 (0.974, 1.717) | 0.076 | 1.370 (0.971, 1.932) | 0.073 |
| AFP, ng/ml (≥400 vs <400） | | 1.118 (0.916, 1.365) | 0.272 | 1.051 (0.834, 1.324) | 0.674 |
| Tumor diameter, cm (≥5 vs <5） | | 5.394 (4.329, 6.721) | <0.001 | 5.675 (4.373, 7.365) | <0.001 |
| Tumor number (Multiple vs Single) | | 1.759 (1.418, 2.184) | <0.001 | 1.767 (1.349, 2.315) | <0.001 |
| Postoperative adjuvant | LR | Reference |  | Reference |  |
|  | PA-TACE | 0.512 (0.411, 0.637) | <0.001 | 0.600 (0.468, 0.770) | <0.001 |
|  | PA-HAIC | 0.476 (0.344, 0.657) | <0.001 | 0.516 (0.347, 0.767) | 0.001 |
|  | PA-Targeted immunotherapy | 0.409 (0.286, 0.585) | <0.001 | 0.524 (0.352, 0.779) | 0.001 |
| Hepatitis (Positive vs negative) | | 0.887 (0.663, 1.185) | 0.416 | 0.934 (0.683, 1.278) | 0.670 |
| Liver cirrhosis (Positive vs negative) | | 0.997 (0.808, 1.181) | 0.806 | 1.023 (0.821, 1.274) | 0.841 |
| Child-pugh grade (B vs A) | | 1.280 (0.572, 2.868) | 0.548 | 3.403 (1.264, 9.163) | 0.015 |
| ALBI grade (2 vs 1) | | 1.182 (0.971, 1.439) | 0.096 | 1.102 (0.880, 1.379) | 0.398 |
| Resection pattern, (Nonanatomic vs Anatomic) | | 1.068 (0.828, 1.378) | 0.611 | 1.158 (0.860, 1.560) | 0.334 |
| BCLC grade (B vs 0+A) | | 2.049 (1.632, 2.573) | <0.001 | 1.791 (1.356, 2.366) | <0.001 |
| Differentiation (Low vs High and/or moderate） | | 2.812 (2.242, 3.527) | <0.001 | 3.131 (2.371, 4.136) | <0.001 |
| MVI (Positive vs negative) | | 2.145 (1.747, 2.634) | <0.001 | 2.043 (1.616, 2.583) | <0.001 |
| Hb, g/L (<132 vs ≥132) | | 0.972 (0.801 vs 1.181) | 0.778 | 0.991 (0.791, 1.242) | 0.937 |
| NLR (≥2.2 vs <2.2) | | 1.339 (1.103, 1.625) | 0.003 | 1.231 (0.985, 1.540) | 0.068 |
| PLR (≥50 vs <50) | | 0.862 (0.711, 1.044) | 0.129 | 0.801 (0.641, 1.003) | 0.053 |
| Total protein, g/l (<70 vs ≥70) | | 1.010 (0.836, 1.220) | 0.919 | 0.972 (0.781, 1.210) | 0.801 |
| ALT, U/L (≥35 vs <35) | | 0.987 (0.817, 1.193) | 0.894 | 0.876 (0.703, 1.090) | 0.235 |
| GGT, U/L (≥60 vs <60) | | 1.162 (0.962, 1.403) | 0.120 | 1.069 (0.860, 1.330) | 0.546 |

PSM, propensity score matching; AFP, alpha-fetoprotein; PA, postoperative adjuvant; LR, liver resection; TACE, transcatheter arterial chemoembolization; HAIC, hepatic artery perfusion chemotherapy; ALBI, albumin-bilirubin; BCLC, barcelona clinic liver cancer; MVI, microvascular invasion; NLR, neutrophil-to-lymphocyte ratio, PLR, platelet-to-lymphocyte ratio, ALT, alanine aminotransferase; GGT, γ-Glutamyltransferase

Supplementary table 2.B Univariate COX Regression Analysis of OS in the Entire Cohort and the PSM Cohort.

| Characteristics | | the Entire cohort | | the PSM cohort | |
| --- | --- | --- | --- | --- | --- |
|  |  | HR (95% CI) | p | HR (95% CI) | p |
| Tumor location, cm (≤2 vs >2) | | 1.518 (1.150, 2.005) | 0.003 | 1.390 (1.005, 1.923) | 0.046 |
| Age, yr（≥56 vs <56） | | 0.897 (0.678, 1.187) | 0.446 | 1.089 (0.788, 1.504) | 0.606 |
| Sex（Male vs Female） | | 1.577 (0.992, 2.506) | 0.054 | 1.563 (0.899, 2.716) | 0.113 |
| AFP, ng/ml (≥400 vs <400） | | 1.277 (0.958, 1.702) | 0.096 | 1.111 (0.794, 1.554) | 0.538 |
| Tumor diameter, cm (≥5 vs <5） | | 4.189 (3.092, 5.674) | <0.001 | 4.035 (2.849, 5.716) | <0.001 |
| Tumor number (Multiple vs Single) | | 1.485 (1.073, 2.054) | 0.017 | 1.381 (0.919, 2.075) | 0.120 |
| Postoperative adjuvant | LR | Reference |  | Reference |  |
|  | PA-TACE | 0.744 (0.546, 1.013) | 0.060 | 1.012 (0.706, 1.452) | 0.947 |
|  | PA-HAIC | 1.103 (0.688, 1.769) | 0.683 | 1.429 (0.803, 2.542) | 0.224 |
|  | PA-Targeted immunotherapy | 1.064 (0.609, 1.860) | 0.827 | 1.605 (0.880, 2.928) | 0.123 |
| Hepatitis (Positive vs negative) | | 1.020 (0.671, 1.551) | 0.926 | 1.180 (0.729, 1.910) | 0.501 |
| Liver cirrhosis (Positive vs negative) | | 0.955 (0.722, 1.263) | 0.746 | 1.061 (0.768, 1.465) | 0.721 |
| Child-pugh grade (B vs A) | | 1.803 (0.575, 53655) | 0.312 | 2.354 (0.582. 9.525) | 0.283 |
| ALBI grade (2 vs 1) | | 0.942 (0.702, 1.264) | 0.690 | 0.798 (0.568, 1.121) | 0.194 |
| Resection pattern, (Nonanatomic vs Anatomic) | | 1.177 (0.827, 1.676) | 0.365 | 1.143 (0.753, 1.735) | 0.529 |
| BCLC grade (B vs 0+A) | | 1.671 (1.194, 2.339) | 0.003 | 1.409 (0.927, 2.140) | 0.108 |
| Differentiation (Low vs High and/or moderate） | | 2.146 (1.575, 2.924) | <0.001 | 1.831 (1.241, 2.702) | 0.002 |
| MVI (Positive vs negative) | | 2.767 (1.993, 3.841) | <0.001 | 2.774 (1.908, 4.033) | <0.001 |
| Hb, g/L (<132 vs ≥132) | | 0.892 (0.669, 1.189) | 0.437 | 0.787 (0.561, 1.104) | 0.165 |
| NLR (≥2.2 vs <2.2) | | 1.318 (0.986, 1.762) | 0.062 | 1.353 (0.964, 1.899) | 0.080 |
| PLR (≥50 vs <50) | | 0.922 (0.694, 1.224) | 0.573 | 0.976 (0.704, 1.354) | 0.886 |
| Total protein, g/l (<70 vs≥70) | | 1.039 (0.787, 1.372) | 0.789 | 1.050 (0.762, 1.449) | 0.764 |
| ALT, U/L (≥35 vs <35) | | 1.059 (0.801, 1.398) | 0.688 | 0.925 (0.671, 1.276) | 0.636 |
| GGT, U/L (≥60 vs <60) | | 0.954 (0.722, 1.260) | 0.738 | 0.871 (0.631, 1.202) | 0.400 |

PSM, propensity score matching; AFP, alpha-fetoprotein; PA, postoperative adjuvant; LR, liver resection; TACE, transcatheter arterial chemoembolization; HAIC, hepatic artery perfusion chemotherapy; ALBI, albumin-bilirubin; BCLC, barcelona clinic liver cancer; MVI, microvascular invasion; NLR, neutrophil-to-lymphocyte ratio, PLR, platelet-to-lymphocyte ratio, ALT, alanine aminotransferase; GGT, γ-Glutamyltransferase

Supplementary table 3.A Validation of the Cox regression analysis results for RFS

| Characteristics | | the Entire cohort | | the PSM cohort | |
| --- | --- | --- | --- | --- | --- |
|  |  | HR (95% CI) | p | HR (95% CI) | p |
| Tumor location, cm (≤2 vs>2) | | 1.897 (1.557, 2.310) | <0.001 | 1.754 (1.396, 2.202) | <0.001 |
| Tumor diameter, cm (≥5 vs <5） | | 4.665 (3.695, 5.889) | <0.001 | 4.984 (3.777, 6.576) | <0.001 |
| Tumor number (Multiple vs Single) | | 2.074 (1.658, 2.594) | <0.001 | 2.083 (1.578, 2.748) | <0.001 |
| PA-treatment regimens | LR | Reference |  | Reference |  |
|  | PA-TACE | 0.483 (0.385, 0.607) | <0.001 | 0.496 (0.381, 0.644) | <0.001 |
|  | PA-HAIC | 0.378 (0.270, 0.528) | <0.001 | 0.356 (0.236, 0.537) | <0.001 |
|  | PA-Targeted immunotherapy | 0.510 (0.356, 0.730) | <0.001 | 0.536 (0.360, 0.798) | 0.002 |
| Differentiation (Low vs High and/or moderate) | | 2.389 (1.872, 3.047) | <0.001 | 2.525 (1.879, 3.394) | <0.001 |
| MVI (positive vs negative) | | 1.556 (1.242, 1.949) | <0.001 | 1.454 (1.121, 1.885) | 0.005- |

PSM, propensity score matching; PA, postoperative adjuvant; LR, liver resection; TACE, transcatheter arterial chemoembolization; HAIC, hepatic artery perfusion chemotherapy; MVI, microvascular invasion.

Supplementary table 3.B Validation of the Cox regression analysis results for OS

| Characteristics | the Entire cohort | | the PSM cohort | |
| --- | --- | --- | --- | --- |
|  | HR (95% CI) | p | HR (95% CI) | p |
| Tumor location，cm (≤2 vs＞2) | 1.707 (1.288, 2.262) | <0.001 | 1.730 (1.241, 2.410) | <0.001 |
| Tumor diameter, cm (≥5 vs ＜5） | 3.743 (2.725, 5.142) | <0.001 | 3.685 (2.546, 5.355) | <0.001 |
| Tumor number (Multiple vs Single) | 1.442 (1.041, 1.997) | 0.028 | - | - |
| MVI (positive vs negative) | 2.072 (1.469, 2.923) | <0.001 | 2.015 (1.359, 2.989) | <0.001 |

PSM, propensity score matching; MVI, microvascular invasion.

Supplementary table 4 Comparative efficacy of identical postoperative adjuvant therapies between central tumor cohort and peripheral tumor cohort

| Characteristics | | | mRFS (months) | | 1-year RFS rate (%) | | 2-year RFS rate (%) | | 3-year RFS rate (%) | | 4-year RFS rate (%) | | 5-year RFS rate (%) | |
| --- | --- | --- | --- | --- | --- | --- | --- | --- | --- | --- | --- | --- | --- | --- |
| the Entire cohort | LR | Central tumor | 16.00 (12.82, 19.18) | | 63.00 (53.40, 72.60) | | 33.50 (23.50, 43.50) | | 14.80 (6.18, 23.42) | | 11.80 (3.18, 20.42) | | - | |
|  |  | Peripheral tumor | 19.00 (14.26, 23.74) | | 70.80 (63.35, 78.25) | | 42.60 (33.98, 51.22) | | 20.80 (13.35, 28.25) | | 12.40 (4.17, 20.63) | | - | |
|  | PA-TACE | Central tumor | 31.00 (20.92, 41.08) | | 80.10 (72.26, 87.94) | | 55.70 (45.70, 65.70) | | 41.60 (31.41, 51.79) | | 23.70 (14.29, 33.11) | | 8.20 (1.54, 14.86) | |
|  |  | Peripheral tumor | 37.00 (28.94, 45.06) | | 85.10 (78.83, 91.37) | | 65.50 (56.88, 74.12) | | 51.60 (42.39, 60.81) | | 36.70 (27.29, 46.11) | | 22.40 (13.58, 31.22) | |
|  | PA-HAIC | Central tumor | 24.00 (8.96, 39.04) | | 74.40 (59.70, 89.10) | | 47.20 (27.21, 67.19) | | 36.00 (15.62, 56.38) | | - | | - | |
|  |  | Peripheral tumor | 41.00 (28.19, 53.81) | | 88.10 (79.87, 96.33) | | 76.20 (64.83, 87.57) | | 50.40 (34.52,66.28) | | 35.90 (14.73, 57.07) | | 13.50 (0, 35.06) | |
|  | PA-Targeted immunotherapy | Central tumor | 27.50 (16.70, 38.30) | | 82.70 (70.16, 95.24) | | 50.80 (33.16, 68.44) | | 31.20 (8.86, 53.54) | | - | | - | |
|  |  | Peripheral tumor | - | | 90.80 (83.16, 98.44) | | 71.40 (57.88, 84.92) | | 59.60 (42.35, 76.85) | | - | | - | |
| the PSM cohort | LR | Central tumor | 18.00 (15.02, 20.98) | | 68.69 (58,80, 78.40) | | 37.90 (27.12, 48.68) | | 16.70 (7.01, 26.30) | | 13.40 (3.60, 23.20) | | - | |
|  |  | Peripheral tumor | 22.50 (16.47, 28.53) | | 73.40 (64.38, 82.42) | | 44.90 (44.12, 55.69) | | 24.40 (14.80, 34.00) | | 14.90 (4.51, 25.29) | | - | |
|  | PA-TACE | Central tumor | 33.50 (25.57, 41.43) | | 85.70 (78.45, 92.95) | | 59.00 (48.61, 69.39) | | 43.80 (33.02, 54.58( | | 25.80 (15.80, 35.80) | | 9.00 (1.94, 16.06) | |
|  |  | Peripheral tumor | 32.50 (27.52, 37.48) | | 83.50 (74.88, 92.14) | | 64.44 (53.23, 75.37) | | 43.10 (31.34, 54.86) | | 27.30 (16.13, 38.47) | | 18.40 (7.82, 28.98) | |
|  | PA-HAIC | Central tumor | 20.00 (2.20, 37.80) | | 69.50 (52.64, 86.36) | | 49.10 (28.32, 69.88) | | 37.40 (16.04, 58.76) | | - | | - | |
|  |  | Peripheral tumor | - | | 86.70 (75.92, 97.48) | | 73.80 (58.90, 88.70) | | 60.50 (39.53, 81.47) | | - | | - | |
|  | PA-Targeted immunotherapy | Central tumor | 26.00 (15.71, 36.29) | | 83.70 (70.57, 96.83) | | 47.00 (28.00, 66.01) | | 26.40 (5.23, 47.57) | | - | | - | |
|  |  | Peripheral tumor | - | | 91.30 (81.89, 100.00) | | 69.40 (52.54, 86.26) | | 54.60 (30.10, 79.10) | | - | | - | |
| Central tumor vs Peripheral tumor | | | p value | | | | | | | | | | |  |
| the Entire cohort | LR | | 0.136 | 0.239 | | 0.170 | | 0.233 | | 0.962 | | - | |  |
|  | PA-TACE | | 0.005 | 0.028 | | 0.126 | | 0.133 | | 0.034 | | 0.002 | |  |
|  | PA-HAIC | | 0.021 | 0.070 | | 0.003 | | 0.136 | | - | | - | |  |
|  | PA-Targeted immunotherapy | | 0.008 | 0.256 | | 0.062 | | 0.008 | | - | | - | |  |
| the PSM cohort | LR | | 0.235 | 0.488 | | 0.322 | | 0.186 | | 0.700 | | - | |  |
|  | PA-TACE | | 0.310 | 0.736 | | 0.484 | | 0.964 | | 0.933 | | 0.058 | |  |
|  | PA-HAIC | | 0.023 | 0.080 | | 0.032 | | 0.047 | | - | | - | |  |
|  | PA-Targeted immunotherapy | | 0.009 | 0.575 | | 0.096 | | 0.019 | | - | | - | |  |

PA, postoperative adjuvant; LR, liver resection; TACE, transcatheter arterial chemoembolization; HAIC, hepatic artery perfusion chemotherapy; PSM, propensity score matching

Supplementary table 5 Comparison of RFS in central tumor cohort and peripheral tumor cohort receiving different postoperative adjuvant therapies

| Characteristics | | mRFS (months) | 1-year RFS rate (%) | 2-year RFS rate (%) | 3-year RFS rate (%) | 4-year RFS rate (%) |  | |  |  |  |  |  |
| --- | --- | --- | --- | --- | --- | --- | --- | --- | --- | --- | --- | --- | --- |
| Central tumor cohort | LR | 16.00 (12.82, 19.17) | 63.00 (55.40, 72.60) | 35.50 (23.50, 43.50) | 14.80 (6.18, 23.42) | 11.80 (3.18, 20.42) |  | |  |  |  |  |  |
|  | PA-TACE | 31.00 (20.92, 41.08) | 80.10 (72.26, 87.94) | 55.70 (45.70, 65.69) | 41.60 (31.41, 51.72) | 23.70 (14.29, 33.11) |  | |  |  |  |  |  |
|  | PA-HAIC | 24.00 (8.96, 39.04) | 74.40 (59.70, 89.10) | 47.20 (27.21, 67.19) | 36.00 (15.62, 56.38) | - |  | |  |  |  |  |  |
|  | PA-Targeted immunotherapy | 24.00 (8.96, 39.04) | 82.70 (70.16. 95.24) | 50.80 (33.16, 68.44) | 31.20 (8.86, 53.53) | - |  | |  |  |  |  |  |
| Peripheral tumor cohort | LR | 19.00 (14.26, 23.74) | 70.80 (63.35, 78.25) | 42.60 (33.98, 51.22) | 20.80 (13.35, 28.25) | 12.40 (4.17, 20.63)- |  | |  |  |  |  |  |
|  | PA-TACE | 37.00 (28.94, 45.06) | 85.10 (78.83, 91.37) | 65.50 (56.88,74.12) | 51.60 (42.39, 60.81) | 36.70 (27.29, 46.11) |  | |  |  |  |  |  |
|  | PA-HAIC | 41.00 (28.19, 53.81) | 88.10 (79.87, 96.33) | 76.20 (64.83, 87.57) | 50.40 (34.52,66.28) | 35.90 (14.73, 57.07) |  | |  |  |  |  |  |
|  | PA-Targeted immunotherapy | - | 90.80 (83.16, 98.44) | 71.40 (57.88, 84.92) | 59.60 (42.35, 76.85) | - |  | |  |  |  |  |  |
| Comparison | | p value | | | | | |  |  |  |  |  |  |
| Central tumor cohort | LR vs PA-TACE | <0.001 | 0.007 | 0.004 | <0.001 | 0.030 |  | |  |  |  |  |  |
|  | LR vs PA-HAIC | 0.062 | 0.187 | 0.193 | 0.008 | - |  | |  |  |  |  |  |
|  | LR vs PA-Targeted immunotherapy | 0.028 | 0.033 | 0.100 | 0.032 | - |  | |  |  |  |  |  |
|  | PA-TACE vs PA-HAIC | 0.411 | 0.479 | 0.368 | 0.498 | - |  | |  |  |  |  |  |
|  | PA-TACE vs PA-Targeted immunotherapy | 0.532 | 0.748 | 0.648 | 0.307 | - |  | |  |  |  |  |  |
|  | PA-HAIC vs PA-Targeted immunotherapy | 0.844 | 0.407 | 0.734 | 0.743 | - |  | |  |  |  |  |  |
| Peripheral tumor cohort | LR vs PA-TACE | <0.001 | 0.004 | <0.001 | <0.001 | <0.001 |  | |  |  |  |  |  |
|  | LR vs PA-HAIC | <0.001 | 0.005 | <0.001 | <0.001 | <0.001 |  | |  |  |  |  |  |
|  | LR vs PA-Targeted immunotherapy | <0.001 | 0.002 | 0.002 | <0.001 | - |  | |  |  |  |  |  |
|  | PA-TACE vs PA-HAIC | 0.552 | 0.530 | 0.166 | 0.876 | 0.883 |  | |  |  |  |  |  |
|  | A-TACE vs PA-Targeted immunotherapy | 0.096 | 0.308 | 0.506 | 0.277 | - |  | |  |  |  |  |  |
|  | PA-HAIC vs PA-Targeted immunotherapy | 0.408 | 0.696 | 0.549 | 0.278 | - |  | |  |  |  |  |  |

PA, postoperative adjuvant; LR, liver resection; TACE, transcatheter arterial chemoembolization; HAIC, hepatic artery perfusion chemotherapy

Supplementary figure 1 All exploratory subgroup analysis for central tumor cohort and peripheral tumor cohort in the PSM cohort


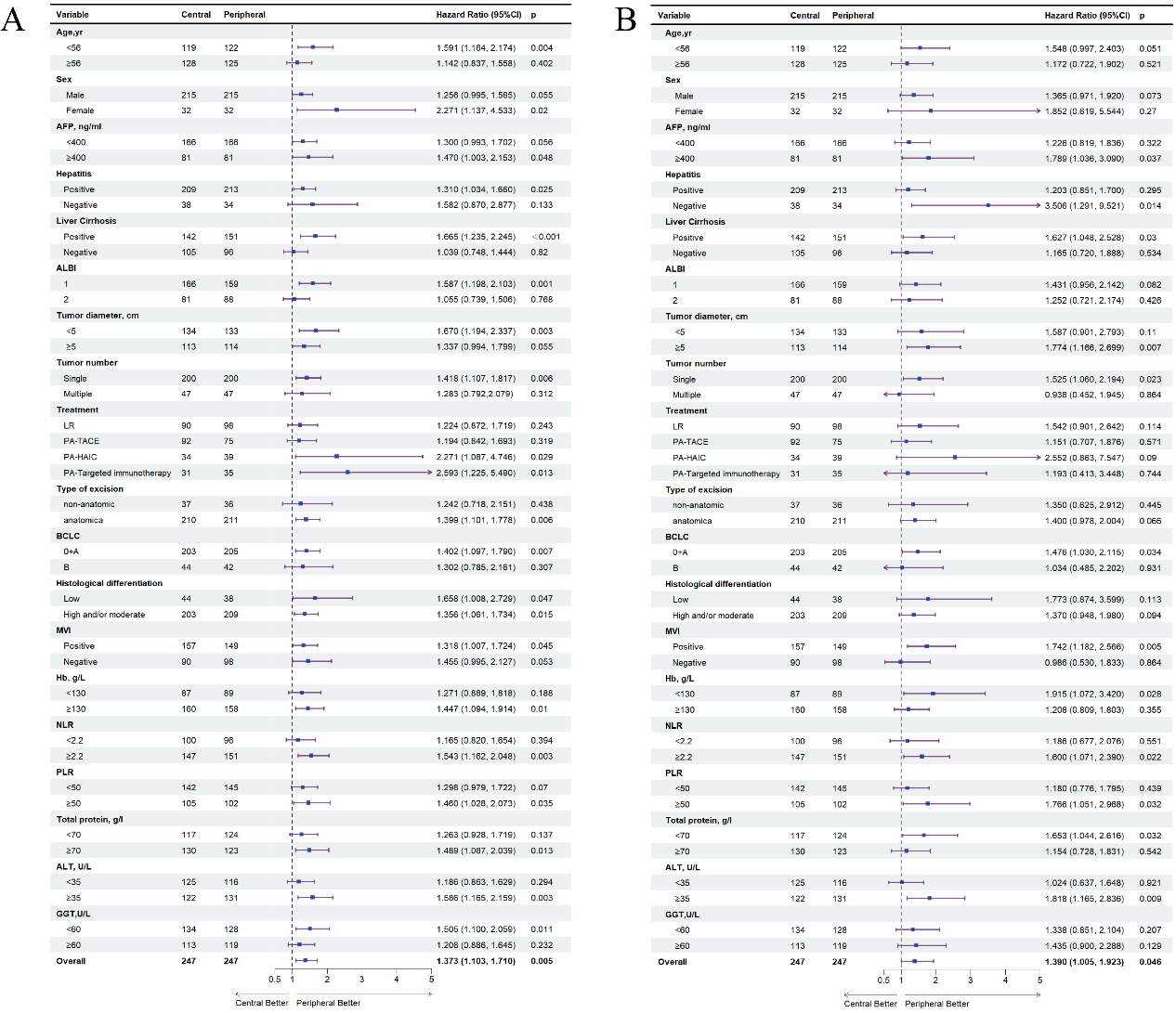


A, All exploratory subgroup analysis about RFS; B, All exploratory subgroup analysis about OS

AFP, alpha-fetoprotein; ALBI, albumin-bilirubin; ALBI grade 1, ≤ -2.60; ALBI grade 2, -2.60 ~ -1.39; PA, postoperative adjuvant; LR, liver resection; TACE, transcatheter arterial chemoembolization; HAIC, hepatic artery perfusion chemotherapy; BCLC, barcelona clinic liver cancer; NLR, neutrophil-to-lymphocyte ratio, PLR, platelet-to-lymphocyte ratio, ALT, alanine aminotransferase, MVI, microvascular invasion; GGT, γ-Glutamyltransferase
